# Editorial: Neuro-immune players of peripheral pain signalling

**DOI:** 10.3389/fimmu.2024.1474270

**Published:** 2024-08-13

**Authors:** Javier Aguilera-Lizarraga, David E. Reed, Philipp Starkl, Yeranddy A. Alpizar

**Affiliations:** ^1^ Department of Pharmacology, University of Cambridge, Cambridge, United Kingdom; ^2^ Gastrointestinal Diseases Research Unit, Queen’s University, Kingston, ON, Canada; ^3^ Research Division of Infection Biology, Department of Medicine I, Medical University of Vienna, Vienna, Austria; ^4^ KU Leuven Department of Microbiology, Immunology and Transplantation, Rega Institute, Division of Virology, Antiviral Drug and Vaccine Research, Laboratory of Molecular Vaccinology and Vaccine Discovery, Leuven, Belgium

**Keywords:** neuroimmune axis, nociceptor, leukocyte, T cell metabolism, exercise, DRG - dorsal root ganglion, astrocytes, pain

Nociceptors comprise a subpopulation of specialised sensory neurons capable of sensing and responding to (potentially) harmful stimuli. This process, termed nociception and often translated into a painful sensation, is essential for the survival and wellbeing of living animals, including humans. However, when pain becomes chronic, it is debilitating and significantly impairs the quality of life. Notably, expression of receptors that recognise immune-derived mediators (1, 2) allow nociceptors to identify cues from the immune system. Indeed, activation of nociceptive nerve fibres by inflammatory molecules leads to the generation of action potentials which are received by the central nervous system and may provoke painful sensation (3). Importantly, immune system perturbations, for instance in response to exogenous agents (4) or after nerve injury (5), can cause aberrant nociceptive signalling and drive the development of chronic pain. All these aspects were within the scope of this Research Topic entitled “Neuro-Immune Players of Peripheral Pain Signalling”. There were two *review* articles, three *original research* articles and one *hypothesis and theory* article published. Starting with the latter, Dong and Ubogu provided preliminary data on the potential role of CD11b^+^CD45^+^ leukocyte infiltration in the sciatic nerve in the context of neuropathic pain. To this end, the authors utilised three mouse models of inflammatory and traumatic peripheral neuropathies (modelling Guillain-Barré syndrome, spontaneous autoimmune peripheral polyneuropathy and sciatic nerve crush), and showed that an increase in CD11b^+^CD45^+^ leukocyte counts in endoneurial sciatic nerves was associated with an impairment in the severity of motor progression. Interestingly, treatment with monoclonal anti-CD11b antibody reduced cold- and heat-induced nociception in their model of traumatic peripheral neuropathy. While it will be important to test the effectiveness of this treatment in other models, these data suggest that targeting CD11b could be developed into a treatment option for patients suffering from neuropathic pain. Further research addressing this possibility is hence warranted.

The two reviews used different approaches. Koop et al. opted for a systematic review with meta-analysis of animal studies to assess the effects of pre-injury exercise on neuroimmune and other physiological and behavioural responses following experimentally induced traumatic peripheral neuropathy. The authors discussed existing evidence and suggest that pre-injury exercise may potentially confer neuroprotection and exert immunoregulatory effects, limiting the sequelae of the nerve injury. However, the authors highlighted the need for caution, as most findings are based on single studies, and insisted on the need for more research to reinforce such evidence. On the other hand, Dou et al. addressed the effects of peripheral nerve damage on T cell metabolism. They first discussed the function and metabolism of T cells in neuropathic pain and how this is influenced by changes in the microenvironment during nerve injury and contribute to pain development. In the second part, the authors focused on T cell metabolism-related molecules and the associated mechanisms that alleviate or facilitate the development of neuropathic pain.

Next, we organised the three original research papers in this Editorial from clinical observational analyses (one study) to basic research using animal models (two studies). In the former, Tang et al. used the large-scale UK Biobank database of genome-wide association study (GWAS) to investigate the causal relationship between autoimmune diseases and chronic pain, including multisite chronic pain and chronic widespread pain. They observed that multisite chronic pain was associated with a higher risk of multiple sclerosis and rheumatoid arthritis, while no significant effects were observed on other autoimmune diseases. Moreover, the authors found that increased body mass index may mediate this effect in rheumatoid arthritis. Further studies are required to clarify the molecular pathophysiological mechanisms of this relationship and the role of increased body weight in the interaction between the immune and the somatosensory nervous systems.

Ballon Romero et al. showed that multiple dental pulp injuries (MDPI), but not a single one, trigger persistent orofacial pain in mice. Animals subjected to MDPI showed signs of dental pain (assessed by mechanical head withdrawal evoked with von Frey filaments) up to 70 days post-injury. These mice exhibited strong and long-term GFAP immunoreactivity in the brainstem, indicative of astrocyte activation. This was accompanied by prolonged alterations in the glutamate-glutamine cycle, apoptotic signalling, and altered GABAergic inhibitory synaptic transmission. Of interest, the effects of MDPI-induced orofacial pain were partially alleviated following 12 sessions of electroacupuncture stimulation, which also reversed astrocytic activation and pro-apoptotic markers, and re-established the GABAergic neurotransmission. Hence, this study proposes that electroacupuncture may exert prolonged analgesic and neuroprotective effects in persistent dental pain in mice, potentially involving the modulation of neuron-glia crosstalk mechanisms.

Finally, Yadav et al. sought to investigate the mechanisms by which lysozyme, a small cationic protein that exerts antibacterial activity through its ability to hydrolyse peptidoglycans in the bacterial cell wall, triggers pain and how this influences neuroinflammation. The authors showed that injection of lysozyme into the paw of mice did not cause inflammation but induced robust pain responses dependent on TLR4 activation. Lysozyme activated TLR4 in both neuronal and immune cells, but the specific activation of Toll/IL-1 receptor domain-containing adaptor inducing interferon-β (TRIF) signalling provokes pain sensitisation without a significant release of inflammatory cytokines. Interestingly, lysozyme increased glutamate concentrations in neuronal cell cultures through TLR4 activation leading to overexpression of mitochondrial glutamate oxaloacetate transaminase 2 (GOT2). Thus, activation of TLR4 by lysozyme through the selective activation of TRIF pathway can induce pain in the absence of inflammation.

In conclusion, this Research Topic included one *hypothesis and theory* article, two *review* articles and three *original research* articles, focusing on the interactions between the immune and the nervous systems in the context of pain. The articles addressed mechanisms involving the role of leukocytes and leukocyte-derived enzymes, T cell-metabolism, exercise and body mass index, DRG signalling pathways and astrocytes focusing on neuro-immune interactions in pain signalling.
